# Management von Patienten mit Tracheostoma während der COVID-19-Pandemie: Literaturüberblick und Demonstration

**DOI:** 10.1007/s00106-020-00892-3

**Published:** 2020-06-08

**Authors:** J. S. Kempfle, H. Löwenheim, M. J. Huebner, H. Iro, S. K. Mueller

**Affiliations:** 1 Abteilung für Hals-Nasen-Ohren-Heilkunde, Universitätsklinikum Tübingen, Tübingen, Deutschland; 2 Abteilung für Kinderkardiologie, Friedrich-Alexander-Universität Erlangen-Nürnberg (FAU), Erlangen, Deutschland; 3 Abteilung für Hals-Nasen-Ohren-Heilkunde, Kopf- und Halschirurgie, Friedrich-Alexander-Universität Erlangen-Nürnberg (FAU), Waldstraße 1, 1054 Erlangen, Deutschland; 4 Department of Otolaryngology, Massachusetts Eye and Ear Infirmary, Boston, MA USA; 5 Harvard Medical School, Boston, MA USA

**Keywords:** Tröpfchen, COVID-19, Aerosole, Kanülenwechsel, PSA, Droplets, COVID-19, Aerosols, Tracheostomy tube change, PPE

## Abstract

**Hintergrund:**

Seit dem Auftreten des neuen Coronavirus im Dezember 2019 in China haben viele Länder Schwierigkeiten, die ansteigende Zahl der Infektionen, auch innerhalb des medizinischen Personals, zu kontrollieren. Es hat sich mittlerweile deutlich gezeigt, dass das neue SARS-CoV-2-Virus insbesondere über Aerosole und Tröpfchen der oberen Atemwege übertragen wird und die Infektionsgefahr bei oberen Atemwegsprozeduren deutlich erhöht ist. Ein Anteil der schwererkrankten beatmungspflichtigen Patienten benötigt ab einem gewissen Zeitpunkt eine Tracheotomie zur langfristigen Beatmung und einfacheren Entwöhnung von der Beatmungsmaschine. Diese Patienten erfordern jedoch im Anschluss eine nicht unerhebliche Betreuung durch medizinisches Pflegepersonal, und es ist bislang unklar, inwieweit die Tracheostomapflege ein Risiko für sekundäre Infektionen darstellt.

**Fragestellung:**

Evaluierung der Gefahr der Tröpfchenbildung bei Trachealkanülenwechsel, Überblick zum Kanülenwechsel bei COVID-19-Patienten.

**Material und Methoden:**

Literaturrecherche, quantitative und qualitative Analyse der Tröpfchenfreisetzung bei Kanülenwechsel an *n* = 8 Patienten, Übersicht und Checkliste für Kanülenwechsel.

**Ergebnisse:**

Diese Studie demonstriert, dass beim Kanülenwechsel, insbesondere bei Einführen der neuen Kanüle, eine nicht unbeträchtliche Menge an Tröpfchen entstehen kann. Eine Aerosolbildung von Partikeln kleiner als 5 µm wurde nicht untersucht.

**Schlussfolgerung:**

Unsere Ergebnisse im Zusammenhang mit der aktuellen Literatur verdeutlichen, dass die Pflege nach Tracheotomie eine hoch risikoreiche Prozedur darstellt und nur von einer kleinen Gruppe von geschultem und gut geschütztem Personal durchgeführt werden sollte.

## Abstract (en)

**Background:**

Since emergence of the new coronavirus in China in December 2019, many countries have been struggling to control skyrocketing numbers of infections, including among healthcare personnel. It has now been clearly demonstrated that SARS-CoV‑2 resides in the upper airways and transmits easily via aerosols and droplets, which significantly increases the risk of infection when performing upper airway procedures. Ventilated COVID-19 patients in a critical condition in the intensive care unit may require tracheotomy for long-term ventilation and to improve weaning. However, the risk of secondary infection of medical personnel performing subsequent tracheostomy care remains unclear.

**Objective:**

This study aimed to evaluate the risk of droplet dispersion during tracheostomy tube change and overview tracheostomy tube change in COVID-19 patients.

**Materials and methods:**

The current literature was reviewed, quantitative and qualitative analyses of droplet formation during tracheostomy tube change in *n* = 8 patients were performed, and an overview of and checklist for tracheostomy tube change were compiled.

**Results:**

This study demonstrates that tracheostomy tube change, in particular insertion of the new tube, may cause significant droplet formation. The aerosolization of particles smaller than 5 µm was not analyzed.

**Conclusion:**

Our data, together with the current literature, clearly emphasize that tracheostomy care is associated with a high infection risk and should only be performed by a small group of well-trained, maximally protected healthcare personnel.

Etwa 5–7 % der Patienten mit einer SARS-CoV-2-Infektion haben einen schweren Verlauf bis hin zu Lungen- und Multiorganversagen und benötigen bei Langzeitintubation gegebenenfalls eine Tracheotomie. Wir demonstrieren in diesem Zusammenhang die Entstehung und Gefahr der Tröpfchenbildung bei Trachealkanülenwechsel und der Trachealkanülenpflege und bieten eine praktische Übersicht zu Vorsichtsmaßnahmen bei der weiterführenden Behandlung von tracheotomierten Patienten.

Die durch das neue Coronavirus, auch Severe-Acute-Respiratory-Syndrome-Coronavirus 2, kurz SARS-CoV‑2 genannt, ausgelöste Erkrankung (COVID-19), zeichnet sich durch eine hohe Variabilität an Symptomen aus. Während eine große Anzahl an SARS-CoV-2-positiven Patienten keine oder nur leichte Symptome einer oberen Atemwegsinfektion hat, gibt es eine Gruppe von Patienten, die eine deutliche Einschränkung ihrer Lungenfunktion mit Pneumonie bis hin zur lebensbedrohlichen Variante mit akutem Lungenversagen („acute respiratory distress syndrome“, ARDS) entwickelt [18, 37, 40]. Mit weltweiter Ausbreitung der COVID-19-Pandemie wird auch die Notwendigkeit von Eingriffen am Atemweg von Patienten zunehmend zum Thema. Dabei hat sich gezeigt, dass die Infektiosität des neuen SARS-CoV‑2 deutlich höher ist als die der Coronaviren der ursprünglichen SARS(Severe Acute Respiratory Syndrome)- oder MERS(Middle East Respiratory Syndrome)-Pandemien und die Viruslast im oberen Respirationstrakt besonders hoch ist [32, 40, 48]. SARS-CoV‑2 wurde von der Bundesanstalt für Arbeitsschutz und Arbeitsmedizin als Erreger der Risikogruppe 3 eingestuft und der Schutzstufe 3 zugeordnet [5]. Nach den Technischen Regeln für Biologische Arbeitsstoffe (TRBA), die vom Ausschuss für Biologische Arbeitsstoffe (ABAS) ermittelt bzw. angepasst und vom Bundesministerium für Arbeit und Soziales bekannt gegeben werden, ergibt sich nach der TRBA 250 „Biologische Arbeitsstoffe im Gesundheitswesen und in der Wohlfahrtspflege“ eine Zuordnung zur Schutzstufe 3 (biologische Schutzstufe, „biosafety level“, BSL), wenn biologische Arbeitsstoffe der Risikogruppe 3 vorliegen. Diese können bereits in niedriger Konzentration eine Infektion bewirken. Hohe Konzentrationen von biologischen Arbeitsstoffen der Risikogruppe 3 können auftreten, wenn Tätigkeiten durchgeführt werden, die eine Übertragung möglich machen, z. B. die Gefahr von Aerosol- und Tröpfchenbildung. Dies gilt auch, wenn nur ein entsprechender Verdacht besteht [31]. Eine Zuordnung zur Schutzstufe 3 bedeutet ein gesteigertes Risiko für Sekundärinfektionen vom Patienten auf das medizinische Personal, sei es durch direkten oder indirekten Kontakt mit dem Patienten. Dazu kommt bei COVID-19 eine steigende Zahl an beatmungspflichtigen Patienten, sodass auch Personal mit Patienten in Kontakt kommt, welches nur wenig oder unzureichende Erfahrung mit Atemwegsmanagement und den assoziierten Risiken hat [16]. Atemwegsmanipulation bei Trachealkanülenwechsel am wachen oder beatmeten Patienten sind gängige Praxis auf COVID-19-Stationen und erfordern spezielle Vorsichtsmaßnahmen mit entsprechender Schulung des Personals [35, 45]. Erste Studien zeigen deutlich die erhöhte Gefahr der Ansteckung bei „aerosolisierenden“ Prozeduren der oberen Atemwege [22, 25, 30, 42]: Damit ist die Freisetzung von kleinsten virusenthaltenden Flüssigkeitspartikeln, im Fall von COVID-19 aus der Schleimhaut des Nasen- und Rachenraums, in die Umgebungsluft gemeint. In dieser Arbeit wird wie folgt differenziert: Eine mechanische Irritation führt zur Bildung von Tröpfchen, („droplets“; aerodynamischer Durchmesser über 5 µm), sowie Feinpartikelaerosolen (aerodynamischer Durchmesser unter 5 µm) [34, 40, 43]. Im Gegensatz zur Übertragung durch Tröpfchen findet die aerogene Übertragung durch Tröpfchenkerne (≤5 μm; Aerosole; „droplet nuclei“) statt. Aerosole gelangen mit der Atemluft direkt in die tiefen Atemwege und umgehen damit wichtige physikalische und immunologische Barrieren der oberen Atemwege. Aerosole sedimentieren nur sehr langsam und können damit in der Luft schwebend über größere Distanzen verbreitet werden [11]. Voraussetzung für eine aerogene Übertragung ist, dass Mikroorganismen unter diesen Bedingungen über einen gewissen Zeitraum infektiös bleiben (z. B. Masern, Varizellen oder die offene Tuberkulose) [43]. Die Übertragung von SARS-CoV‑2 durch Aerosole ist gut nachvollziehbar, da mittlerweile experimentell nachgewiesen ist, dass das Virus (wie auch SARS-CoV-1) in Aerosolen stundenlang (mind. 3 h) nachweisbar ist [10].

Diese experimentellen Befunde stimmen mit klinischen Beobachtungen bei SARS-CoV-1-Infektionen überein, bei denen diese aerosole Übertragungsform mit nosokomialer Ausbreitung und „super spreading events“ in Verbindung gebracht wurde [41, 46]. Ein Großteil der Erfahrung über das Infektionspotenzial von respiratorischen Viren im Allgemeinen und Coronaviren im Speziellen beruht auf Ergebnissen aus Forschung mit Influenzaviren. Die Gruppe der allgemein bekannten Erkältungs-Coronaviren spielt dabei eine untergeordnete Rolle und wird hauptsächlich im Zusammenhang mit den bereits erwähnten SARS- und MERS-Ausbrüchen der Vergangenheit erwähnt [19]. Allerdings zeigt eine aktuelle Studie aus Deutschland einen deutlichen Unterschied dieser bekannten Erreger zum neuen SARS-CoV‑2 auf: Dieser besteht im Wesentlichen in der Tatsache, dass SARS-CoV‑2 im Gegensatz zu seinen vorherigen Verwandten nicht hauptsächlich in den unteren Atemwegen und der Lunge repliziert wird, sondern zunächst vor allem von der Schleimhaut der oberen Atemwege [40]. Die dort gefundene Viruskonzentration war mehr als tausendfach höher verglichen mit MERS- oder SARS-Coronaviren [19]. Zudem fand die unabhängige Hauptreplikation des aktiven Virus in der Schleimhaut der oberen Atemwege während des Prodromal- und Frühstadiums der Infektion statt [40]. Dabei zeigte sich der Höhepunkt der Virusausscheidung vor der eigentlichen Symptomphase, was eine Detektion von potenziell infizierten Patienten zusätzlich erschwert. All dies im Zusammenhang mit einer protrahierten Virusausscheidung in Sputum, Tröpfchen und Aerosolen bis in die Spätphase der Erkrankung kompliziert die stationäre Patientenbehandlung [38] und erklärt Berichte und Veröffentlichungen, welche eine besonders hohe Anzahl an Neuinfektionen bei medizinischem Personal nach operativen SARS-CoV-2-Atemwegsprozeduren beschrieben hatten [25].

Eine kürzlich in *Nature Medicine* erschienene Studie, welche sich mit den Erkältungs-Coronaviren beschäftigte, konnte diese sowohl in der ausgeatmeten Luft über einen Zeitraum von 30 min als auch in erhöhter Konzentration in Aerosolen und Tröpfchen von wiederholt hustenden Patienten nachweisen [19]. Ob Tröpfchen von hustenden Patienten mehr Virus enthalten als Aerosole der Ausatemluft, wird jedoch kontrovers diskutiert [15, 42]. In diesem Zusammenhang haben Bleier et al. auch die Generierung von Tröpfchen und Aerosolen über die Nase sowie bei endonasalen Eingriffen in Kadavern untersucht und fanden dabei, dass sowohl Husten als auch Niesen Tröpfchen und Aerosole in einem Radius von über 60 cm streuen konnte [42]. Manipulation mit Bohrern, wie etwa bei Nasennebenhöhleneingriffen, erzeugte eine deutliche Tröpfchendispersion, wohingegen kalte Instrumentation nur minimale Aerosolbildung zeigte [42].

Die hohe Effizienz, mit der sich SARS-CoV‑2 im oberen Atemweg repliziert und über Aerosole und Tröpfchen ausgeschieden wird, erfordert entsprechende Schutzkleidung, die über die normalen Mundschutzmasken hinausgeht. Die Deutsche Gesellschaft für HNO-Heilkunde, Kopf- und Hals-Chirurgie (DGHNO-KHC) hat kürzlich gemeinsam mit ihrer Arbeitsgemeinschaft Laryngologie und Trachealerkrankungen eine Stellungnahme für direkte Atemwegsinterventionen veröffentlicht [20]. Nach Empfehlung des Robert Koch-Instituts (RKI; Stand: 24.04.2020) müssen bei der direkten Versorgung von Patienten mit bestätigter oder wahrscheinlicher COVID-19 gemäß den Arbeitsschutzvorgaben mindestens FFP2-Masken getragen werden (Biostoffverordnung in Verbindung mit der TRBA 250) [28]. Die spezifische Situation von operativen Eingriffen im oberen Respirationstrakt einschließlich der Tracheotomie ist in den Empfehlungen des RKI nicht abgebildet. Nach Beschluss 609 der ABAS (06/2012) sind „FFP3-Masken bei Tätigkeiten, bei denen das Husten des Patienten provoziert wird, z. B. während einer Bronchoskopie, Intubation oder beim Absaugen, zu tragen“ [2]. Nach aktueller Empfehlung der BAuA vom 27.03.2020 werden FFP3-Masken „z. B. für Tätigkeiten an Patienten, die stark husten oder zum Husten provoziert werden“ empfohlen [6]. (Anmerkung: Das RKI ist für den Arbeitsschutz nicht zuständig, sondern „nur“ für den allgemeinen Infektionsschutz. Zuständig für den Arbeitsschutz ist das Bundesamt für Arbeitsschutz und Arbeitsmedizin; BAuA). Die operative Tätigkeit am oberen Respirationstrakt stellt eine direkte Intervention in einem hoch belasteten Bereich dar und führt regelmäßig zur Tröpfchen- und Aerosolbildung. Als Standard sollte daher eine FFP3-Maske mit Schutzhelm oder Schutzbrille sowie ein flüssigkeitsdichter Kittel getragen werden. Ein Bericht aus Italien warnte allerdings, dass es dort zu Sekundärinfektion beider Operateure nach Tracheotomie mit lediglich FFP3-Masken gekommen war^1^. In der Vergangenheit gab es zudem Berichte über SARS-Infektionen trotz FFP3-Masken nach Wiederbelebungsmaßnahmen bei infizierten Patienten [8]. Daher wird international häufig die Verwendung von gebläseunterstütztem Atemschutz („powered air purifying respirator“, PAPR) für aerosolisierende Prozeduren empfohlen, da diese ein höheres Level an Sicherheit (bis zu BSL-3) gegenüber flüchtigen Pathogenen bieten [29]. Dabei handelt es sich um einen batteriebetriebenen Atemschutz, der Luft über einen High-Efficiency-Particulate-Air(HEPA)-Filter ansaugt und aufreinigt und einem Schutzhelm zuführt, der zusätzlich vor direktem Kontakt mit Aerosolen und Tröpfchen isoliert [12, 29]. Die vom Operateur ausgeatmete, ungereinigte Luft kann jedoch in geringer Menge, je nach PAPR-Haube, aus dem Helm heraustreten, und stellt ein theoretisches Kontaminationsrisiko des sterilen Operationsfelds dar. Es gibt dazu jedoch bisher nur begrenzte Daten [13] und keine abschließenden Studien [14, 29, 30]. Unabhängig vom Atemschutz empfiehlt der ABAS im Zusammenhang mit SARS-CoV‑2 (Stand: 06.04.2020) für längere Tragedauer sowie bei Lieferengpässen von FFP2- und FFP3-Masken aufgrund der deutlich geringeren körperlichen Belastung den Einsatz von gebläseunterstütztem Atemschutz [3]. Nach dieser Empfehlung gewährleistet der Einsatz von gebläseunterstütztem Atemschutz ein hohes Schutzniveau der Beschäftigten und ermöglicht eine einfachere und fehlerfreiere Handhabung als FFP2- und FFP3-Masken. Für FFP-Masken ohne Ausatemventil gilt eine Tragezeitbeschränkung von nur 75 min, für gebläsegestützten Atemschutz besteht keine Tragezeitbeschränkung [6].

Während erste Leitlinien sowohl in Deutschland als auch international vorläufige Empfehlungen zu Vorsichtsmaßnahmen bei In- und Extubationen oder Tracheotomien von beatmungspflichtigen COVID-19-Patienten aussprechen [1, 4, 12, 20, 22, 30, 35], ist vergleichsweise wenig Information zum weiteren Umgang mit einem tracheotomierten SARS-CoV-2-positiven Patienten auf Station, ambulant oder während der Rehabilitation zu finden [10].

Die folgende Arbeit untersuchte das spezielle Risiko der Tröpfchenbildung bei der Tracheostomapflege, speziell bei Kanülenwechsel und Absaugung, und widmet sich insbesondere den Vorsichtsmaßnahmen beim Umgang mit tracheotomierten COVID-19-Patienten [1, 9].

## Methoden

### Patientenkollektiv

Es wurden *n* = 8 SARS-CoV‑2 negative Patienten eingeschlossen, die eine Tracheotomie aufgrund einer otorhinolaryngologischen Operation erhalten hatten. Diese Patienten waren mittels einer Trachealkanüle Größe 9,0 (Tracheoflex, 9,0 mm, mit Cuff, Fa. Rüsch, Berlin, Deutschland) versorgt. Vor den Versuchen erhielten die Patienten wenigstens 12 h keine Inhalation mit einer Ringer-Lösung (oder Gleichwertigem). Die Ethikkommission der Friedrich-Alexander-Universität Erlangen-Nürnberg (FAU) hat der Durchführung dieser Versuche zugestimmt.

### Aufbau des Experiments

**Figure 1:**
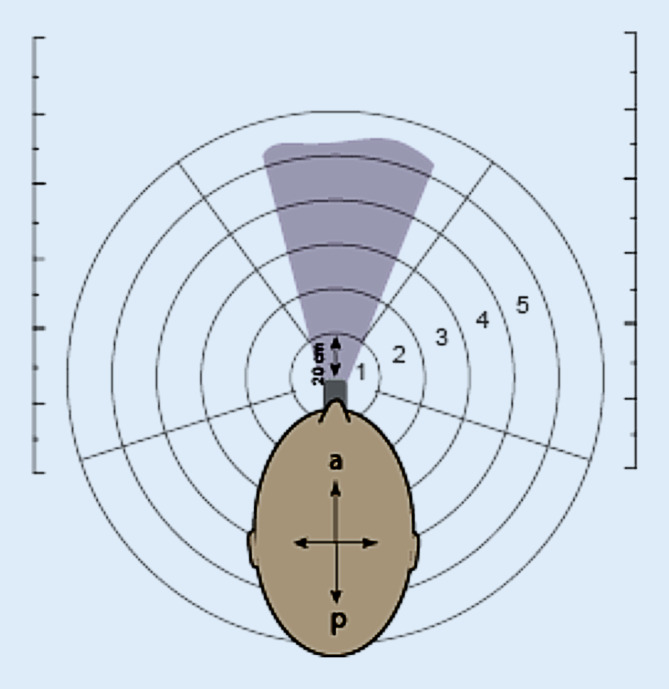

Für die Versuche wurde jeder der tracheotomierten Patienten sitzend positioniert. Vor und unterhalb des Tracheostomas bzw. der Trachealkanüle des Patienten wurde eine weiße Fläche (2 × 2 m) ausgebreitet, die innerhalb von nahezu 360° die Tröpfchen auffangen konnte. Die weiße Fläche wurde in verschiedene kreisförmige Sektionen unterteilt, deren Zentrum das Tracheostoma war. Der Radius der Unterteilungen wurde alle 20 cm von der Öffnung des Tracheostomas des Patienten definiert (Zonen 1–10, Abb. 1).

Zudem wurden 2 verschiedene Patentblau-Lösungen bereitgestellt. Zum einen wurde eine pure Patentblau-Lösung (25 mg/ml, Fa. Guerbet GmbH, Sulzbach, Deutschland) verwendet, zum anderen eine verdünnte Patentblau-Lösung. Um diese herzustellen, wurden 50 ml Trinkwasser mit 3 Tropfen der genannten, unverdünnten Patentblau-Lösung gemischt. Diese Lösung stellt die Standardverdünnung bei unseren postoperativen Schluckversuchen dar.

Alle Versuche wurden vom gleichen Untersucher durchgeführt. Der Untersucher war mit einem Mund- und Augenschutz sowie einem Kittel ausgestattet und erfahren im Umgang mit tracheotomierten Patienten.

### Zielsetzung und Versuchsdurchführung

Die Zielsetzung der Versuche war die Simulation verschiedener routinemäßiger Tätigkeiten bei tracheotomierten Patienten und die Quantifizierung der Tröpfchenbildung in Hinblick auf den Umgang mit COVID-19-Patienten. Hierzu wurden die folgenden Situationen evaluiert: A) Husten mit feuchter Nase (hygroskopischer Kondensatorbefeuchter, Aqua + TS, Fa. Hudson RCI, Wayne/PA, USA); B) Absaugen des Patienten mit einem offenen, flexiblen Absaugsystem mit feuchter Nase; C) Absaugen des Patienten mit einem offenen, flexiblen Absaugsystem ohne feuchte Nase; D) Kanülenwechsel ohne HME-Filter (Wärme- und Feuchtigkeitsaustauscher, Bakterien- und Virenfilter, Teleflex Humid-Vent® filter compact 19401, Wayne, PA, USA); E) Kanülenwechsel mit HME-Filter.

Die Versuche A) bis C) wurden dreifach pro Patient durchgeführt. Für A) bis C) wurden 2 Tropfen der unverdünnten Patentblau-Lösung auf die äußeren 2 cm der Trachealkanüle gegeben. Für A) wurde der Patient aufgefordert zu husten. Für B) und C) wurde der reflektorische Hustenstoß beim Absaugen abgewartet. Für D) und E) wurde die Trachealkanüle entfernt und ein Schluckversuch durchgeführt. Dieser Versuch wurde an den Tagen durchgeführt, an denen die Patienten ohnehin einen Schluckversuch zur Aspirationsevaluation erhielten. Hierzu wurde der Patient aufgefordert, einen Schluck der verdünnten Lösung in den Mund zu nehmen, kurz im Mund zu behalten und dann zu schlucken. Hiernach wurde die Kanüle wiedereingesetzt.

### Auswertung der Versuche

Für alle Versuche wurde beim freiwilligen und induzierten Husten ausgewertet, bis zu welcher Entfernung eine Tröpfchenbildung zu finden war. Außerdem wurde die Anzahl der Tröpfchen pro Unterbezirk sowie die Größe der Tröpfchen gemessen. Zudem wurde die Tröpfchenbildung an der Kleidung und der persönlichen Schutzausrüstung des Untersuchers nach Anzahl und Größe der Tröpfchen quantifiziert.

### Bildbearbeitung und Quantifizierung der Tröpfchengröße

Die Bilder wurden per IPhone-Kamera (IPhone S6, Fa. Apple, Cupertino/CA, USA) aufgenommen und im JPG-Format importiert. ImageJ (Version 2.0.0-rc-69/1.52p) wurde für alle Messungen verwendet. Zuerst wurde das Vorhandensein von Tröpfchen untersucht. Die vorhandenen Tröpfchen wurden nun mit algorithmischer Subtraktion des Hintergrunds untersucht, um reflektierendes Licht zu entfernen („light background“, „separate colours“, „sliding paraboloid“). Die Quantifizierung wurde mittels der Umrechnung von Pixelanzahl und Längenbestimmung der Tröpfchen durchgeführt.

## Ergebnisse

### Quantifizierung der Tröpfchengröße und der Tröpfchenverteilung

**Figure 2:**
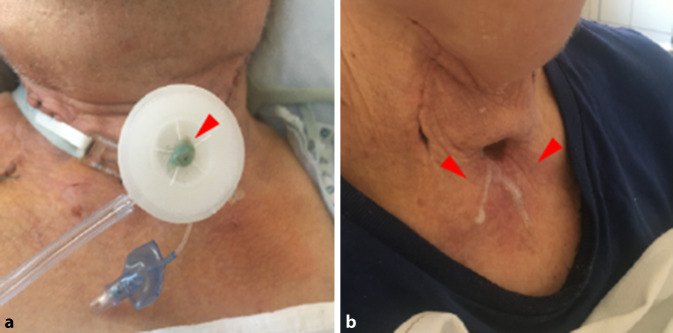

Beim Husten mit feuchter Nase konnten keine Tröpfchen auf der weißen Fläche gefunden werden. Die Innenfläche der feuchten Nase enthielt Tröpfchen von 300 µm bis 5 mm. Bei forciertem Nachhusten des Patienten wurde bei 5/8 Patienten verdicktes Sekret außerhalb der feuchten Nase sichtbar (Abb. 2a), eine Tröpfchenbildung auf der weißen Fläche war nicht zu sehen.

**Figure 3:**
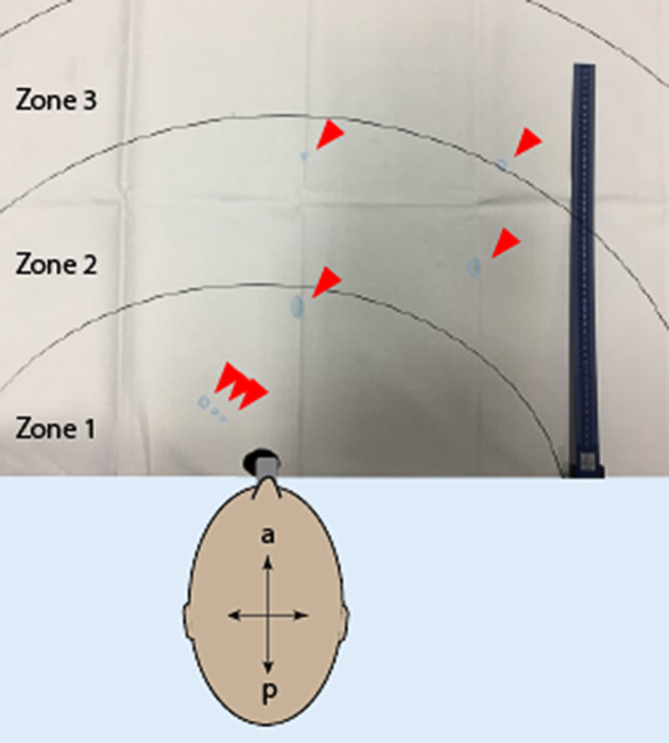

Beim offenen Absaugen wurde darauf geachtet, dass hauptsächlich die Kanüleninnenseite des Patienten und die anliegende Trachea abgesaugt wurde. Bronchiales Absaugen und Manipulationen in der Trachea wurden explizit vermieden. Hierbei konnte keine Tröpfchenbildung mit feuchter Nase gemessen werden. Bei einem Patienten zeigten sich nach Absaugen ohne feuchte Nase 2 Tröpfchen (Entfernung 20 bzw. 30 cm, Größe 3 und 5 mm). Bei Durchführung des Blauschlucks zeigte auch bei reflektorischem Husten keine Tröpfchenbildung, sondern eine Sekretion von relativ flüssigem Sekret aus dem Tracheostoma (Abb. 2b). Bei Wiedereinsetzen der Kanüle kam es zu dem heftigsten Hustenstoß. Hierbei betrug die durchschnittliche Entfernung des Sekrets ohne HME-Filter 65,1 (0–210 cm). Das Sekret war hierbei in den Zonen 1 (25 %), 2 (12,5 %), 3 (12,5 %), 4 (12,5 %), 5 (12,5 %), 6 (12,5 %) und ≥10 (12,5 %) verteilt worden. Bei einem Patienten flog das Sekret über Zone 10 hinaus. Das jeweilige Sekret war von 2 mm bis ca. 2 cm groß (Abb. 3). Beim Einsetzen der Kanüle mit HME-Filter wurde keine Tröpfchenbildung gemessen. Der Untersucher positionierte sich wie bei der standardmäßigen Untersuchung lateral zum Patienten. Auf Brille, Mundschutz und Kittel wurde bei keinem der Versuche eine Tröpfchenbildung festgestellt. Eine Aerosolbildung (kleiner als 5 µm) wurde nicht untersucht.

## Praktische Empfehlungen

### Vorsichtsmaßnahmen und Anleitung gelten für alle Patienten mit:

- Bestätigter SARS-CoV-2-Infektion basierend auf positivem PCR-Test
- Dringendem Verdacht und 1) initial ausstehendem Testergebnis
- Dringendem Verdacht und 2) ausstehendem Folge-Testergebnis
- Dringendem Verdacht und zweifach negativem Nasen- oder Rachenabstrich mit ausstehender Bildgebung der Lunge oder ausstehendem Trachealabstrich

### Kanülenpflege und Kanülenwechsel auf Station

Ziel der Empfehlungen sind die Sicherheit des Patienten sowie die Risikominimierung und Expositionsminimierung für das medizinische Personal sowie die Minimierung des Verbrauchs von PSA (persönliche Schutzausrüstung). Diese praktischen Vorschläge basieren in diesem relativ frühen Stadium der Pandemie auf Literatur und eigenen Erfahrungen. Zu beachten ist jedoch, dass diese der jeweiligen Situation, dem Patienten, dem medizinischen Team und den Ressourcen anzupassen sind (Tab. 1, 2 und 3). Für jegliche Atemwegsintervention an einem beatmeten COVID-19-Patienten spielen ein angemessener Ort und eine angemessene Zeit eine große Rolle und sollte gut überlegt werden. Idealerweise befinden sich Patienten in Negativdruck-Räumen, die eine gewisse Bewegungsfreiheit um das Patientenbett und die Beatmungsmaschine ermöglichen. Der Kopf des Patienten sollte aus 3 Richtungen frei zugänglich sein. Benötigte Instrumente sollten vor Betreten des Zimmers zusammengestellt und überprüft werden, um unnötige Wege in und aus dem Zimmer zu vermeiden und um die Zeitdauer am Patientenbett sowie den PSA-Verbrauch so gering wie möglich zu halten. Die eigentliche, aerosolisierende Prozedur sollte so kurz wie möglich gehalten werden und der Kontakt zum Patienten dabei aufs Minimale beschränkt sein. Bei Kanülenwechsel an Patienten, die an einem Beatmungssystem angeschlossen sind und anschließend auch wieder das Beatmungssystem benötigen, sollte ein Anästhesist zugegen sein, um die mechanische Ventilation individuell getaktet kurzfristig auszusetzen.

**Table 1:** 

**Figure 4:**
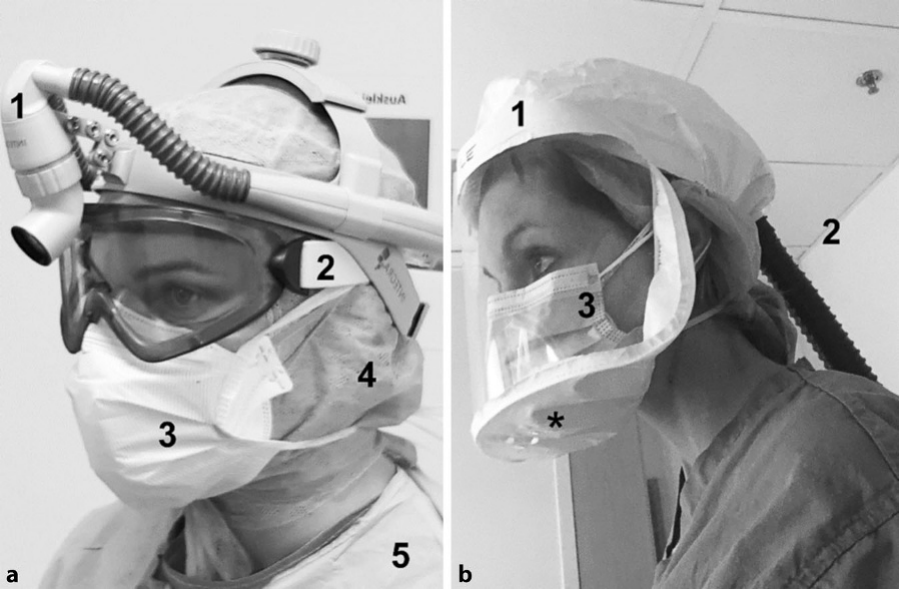

– **Teamauswahl:** So klein wie nötig, so erfahren wie möglich (Ärzteteam HNO und Anästhesie bei beatmeten Patienten, Pflege)
– **Alle:** Anziehen der persönlichen Schutzausrüstung (FFP2- oder FFP3-Maske, chirurgische Maske, 3M-Schutzbrille, Schutzkittel, 2 Paar sterile Handschuhe, Stirnkranz; Abb. 4b)
– **Pflege**: Vorbereitung und Überprüfung der Vollständigkeit aller nötigen Materialien außerhalb des Zimmers
– **Anästhesie**: Sedierung und Relaxierung überprüfen
– **HNO:** Lagerung des Patienten
– **Pflege**: Stellen eines Mülleimers neben den Kopf des Patienten
– **Pflege:** Auslegen der Instrumente, Kanülen, Anschließen der Absaugung
– **Anästhesie**: Präoxygenierung
– **Pflege**: Überprüfen des Cuffs der neuen Kanüle
– **HNO:** Absaugen des Patienten über eine geschlossene Absaugung
– **Anästhesie**: Beatmungspause nach Absprache mit der HNO
– **Pflege**: Entblocken des alten Cuffs
– **HNO:** Entfernung der alten Trachealkanüle samt Gänsegurgel und HME-Filter
– **HNO:** Abwerfen der Trachealkanüle in den bereitgestellten Mülleimer
– **HNO:** Einsetzen der neuen Trachealkanüle mit Gänsegurgel und HME-Filter
– **HNO:** Tracheales Absaugen vermeiden!
– **Pflege**: Blocken des Trachealkanülen-Cuffs
– **Anästhesie:** Anstecken des Beatmungsschlauchs
– **Anästhesie: **Beginn der Beatmung und Verifizierung von CO_2_
– **CO**_**2**_** vorhanden?**
– **Ja** **→** **HNO:** Befestigung der Trachealkanüle mittels Bändchen und Schlitzkompresse
– **Nein** **→** Repositionierung der Kanüle
– **Alle:** Entfernung der persönlichen Schutzausrüstung nach Protokoll (**2. Pflege **kontrolliert)

**Table 2:** 

– **Teamauswahl:** So klein wie nötig, so erfahren wie möglich (Ärzteteam HNO und Anästhesie bei beatmeten Patienten, Pflege)
– **Alle:** Anziehen der persönlichen Schutzausrüstung (FFP2- oder FFP3-Maske, chirurgische Maske, 3M-Schutzbrille, Schutzkittel, 2 Paar sterile Handschuhe, Stirnkranz)
– **Pflege**: Vorbereitung und Überprüfung der Vollständigkeit aller nötigen Materialien außerhalb des Zimmers
– **HNO:** Aufsetzen des Patienten
– **Pflege**: Stellen eines Mülleimers neben den Kopf des Patienten
– **Pflege:** Auslegen der Instrumente, Kanülen, Anschließen der Absaugung
– **Pflege**: Präoxygenierung/Hochstellen des Sauerstoffs (wenn nötig)
– **Pflege**: Überprüfen des Cuffs der neuen Kanüle
– **HNO:** Absaugen des Patienten
– **Pflege:** Entblocken des alten Cuffs unter Absaugen
– **HNO:** Entfernung der alten Trachealkanüle samt Gänsegurgel und HME-Filter
– **HNO:** Abwerfen der Trachealkanüle in den bereitgestellten Mülleimer
– **HNO:** Absaugen des Sekrets am Tracheostoma (Tracheales Absaugen vermeiden!)
– **HNO:** Einsetzen der neuen Trachealkanüle mit Gänsegurgel und HME-Filter
– **Pflege**: Blocken des Trachealkanülen-Cuffs
– **HNO**: Verifizierung der Lage und Durchgängigkeit über die Sättigung, ansonsten Repositionierung notwendig
– **Alle:** Entfernung der persönlichen Schutzausrüstung nach Protokoll (**2. Pflege **kontrolliert)

**Table 3:** 

– Ausziehen der obersten Handschuhe im Zimmer
– Händedesinfektion vor Verlassen des Zimmers
– Schließen der Tür zum Patientenzimmer
– Abwurf von Einmalkittel und Handschuhen
– Händedesinfektion
– Anziehen von neuen Handschuhen
– Ablegen von Schutzbrille/Gesichtsschutzschild/FFP3 an designierter unreiner Station
– Erneute Händedesinfektion und Anlegen von unsterilen Handschuhen
– Desinfektion von Schutzschild oder Schutzbrille entsprechend der Richtlinien. PSA dann in klarer Plastikhülle mit Namen für spätere Verwendung verstauen
– FFP2- oder FFP3-Maske separat in klarer Plastiktüte verstauen und, je nach Richtlinien der Klinik, zur Dekontaminierung übergeben
– Falls PAPR verwendet wurde, sollten Batterie und Schlauch ebenfalls mit Desinfektionstüchern abgewischt und zur weiteren Reinigung übergeben werden

Wann immer möglich, sollte das Absaugen der Kanüle vermieden oder extrem reduziert werden. Ist ein Absaugen unvermeidbar, sollte ein geschlossenes System mit HME-Virus-Filter zur Minimierung der Trachealsekretstreuung verwendet werden.

Ein Kanülenwechsel nach früher Tracheotomie sollte erst nach der zweiten, oder wenn möglich, nach der dritten Infektionswoche erfolgen. Zu diesem Zeitpunkt sollte die Erkrankung abgeklungen und die Viruslast vergleichsweise gering sein [18, 32]. Der HME-Filterwechsel ist je nach Hersteller alle 24 bis 48 h empfehlenswert, dabei gelten die gleichen PSA-Vorsichtsmaßnahmen wie beim Kanülenwechsel.

Im Fall einer akzidentellen Dekanülierung sollte das Tracheostoma sofort abgedeckt werden, um Exposition des Personals zu verringern (Kompresse oder FFP2-Maske).

### Vorsichtsmaßnahmen zur Infektionskontrolle: PSA

Empfohlen wird beim Umgang mit tracheotomierten SARS-CoV-2-positiven Patienten die Verwendung von mindestens FFP2- oder besser FFP3-Masken sowie eines Gesichtsschutzschildes (einfache Schutzbrillen bieten nur unzureichenden Schutz vor seitlich eindringenden Partikeln), eines zusätzlich flüssigkeitsdichten Kittels und doppelten Handschuhen. Wenn vorhanden, bietet ein gebläseunterstützter Atemschutz mit Haube (PAPR) nicht nur höhere Sicherheit, sondern zudem mehr Bewegungsfreiheit und eine deutlich geringere körperliche Belastung sowie eine einfachere und fehlerfreiere Handhabung [3] bei Kanülenwechsel und im Umgang mit agitierten Patienten. Haare sollten zudem unter einer Einmalkopfbedeckung verborgen werden, freiliegende Körperoberflächen, wie an Hals und Handgelenken, abgedeckt und gegebenenfalls mit Klebeband fixiert werden. Wird statt des vollen Gesichtsschutzes nur eine Augenbrille getragen, sollte immer eine normale Mundschutzmaske über der FFP2- oder FFP3-Maske getragen werden, um diese vor Sekretkontamination zu schützen. Bei Verwendung von gebläseunterstütztem Atemschutz (PAPR) mit lose sitzender Haube ist das zusätzliche Tragen der FFP2- oder FFP3-Maske als extra Schutz im Fall eines Batterieversagens empfohlen (Abb. 4).

### Korrektes An- und Ablegen sowie Beseitigung der Schutzkleidung

Unerfahrenes Personal und fehlende Einweisung von Personal können zu falschem Anlegen der Schutzausrüstung führen und das Risiko für Infektionen deutlich erhöhen. Nach erfolgreicher Prozedur und Verlassen des Raums werden korrekte Dekontaminierung und Abfallbeseitigung oft vergessen – ein vermeidbarer Fehler, der sich aber bereits als einer der Hauptgründe für Sekundärinfektionen bei medizinischem Personal mit COVID-19 herausgestellt hat. Um dies zu vermeiden, wird empfohlen, dass entsprechendes Personal vorab im korrekten An- und Ablegen der Masken, des Gesichtsschutzschildes und der Schutzkittel geschult wird. Dabei spielt die Reihenfolge beim An- und Ablegen eine große Rolle, und es empfiehlt sich, dies wiederholt an Kollegen oder SARS-CoV-2-negativen Patienten zu üben. Nach Handhygiene sollte wiederverwendbare PSA noch außerhalb des Zimmers und vor etwaigen Einmalartikeln angelegt werden. Wird optional ein gebläseunterstützter Atemschutz mit Haube (PAPR) verwendet, so sollte der Schutzkittel über die Batterie und den zuführenden Schlauch des Gebläses angezogen werden.

Während die Einmalschutzkleidung vor Verlassen des Patientenzimmers abgelegt werden kann, müssen FFP2/FFP3 und der Gesichtsschutzschild oder die Schutzbrille aufgrund der Ressourcenknappheit oft wiederverwendet werden. Eine designierte „reine“, sowie eine separate „unreine“ Station sollten vor den Zimmern (z. B. auf Patienten-Nachttischen) oder außerhalb des Op.-Saals aufgebaut werden. Die unreine Station dient zur Reinigung der wiederverwendbaren PSA in Abhängigkeit der jeweiligen Herstellerempfehlungen. Wenn möglich, sollte die FFP2- oder FFP3-Maske nach einer solchen, hoch aerosolisierenden Prozedur nicht mehr verwendet werden.

Insgesamt gilt, nur geschultes und erfahrenes Personal sollte in die Behandlung dieser Patienten involviert sein, unerfahrene Assistenzärzte und Pflegepersonal sowie Medizinstudenten sind von diesen Patienten fernzuhalten. Kommunikation und Vorbereitung bestimmen letztendlich das Risiko der Folgeinfektionen.

Ist ein separates Reinigungsteam für Abholung und Dekontaminierung der PSA zuständig, muss potenziell kontaminierte Schutzausrüstung nach vorheriger Absprache so verstaut werden, dass sie kein Infektionsrisiko für weiteres Personal darstellt. Personal, das für Zimmerreinigung und Reinigung der Instrumente und Schutzkleidung zuständig ist, muss ebenfalls mit entsprechender Schutzausrüstung ausgestattet werden. Abwurfbehälter für PSA, Gefahrenabfall und scharfe Gegenstände sollten sowohl im Patientenzimmer als auch direkt vor dem Zimmer vorhanden sein.

## Diskussion

Unsere Literaturrecherche zeigt, dass sowohl die Intubation als auch chirurgische und nichtchirurgische Manipulationen der oberen Atemwege von SARS-CoV-2-positiven Patienten als hoch riskant sowie tröpfchen- und aerosolbildend eingeschätzt werden müssen. Unsere ergänzend durchgeführte Studie verdeutlicht zudem, dass in diese Hochrisikogruppe auch tracheotomierte COVID-19-Patienten im weiteren stationären Verlauf einzuschließen sind, da es mit hoher Wahrscheinlichkeit zur Übertragung von virushaltigen Aerosolen oder Tröpfchen bei Prozeduren wie Kanülenwechsel, Stomapflege und Absaugung kommt.

Studien im Zusammenhang mit der früheren SARS-Infektion sowie aktuell im Zuge der COVID-19-Pandemie verzeichneten eine Ansteckungsrate von ca. 5–20 % bei medizinischem Personal, und eine nicht unbeträchtliche Zahl davon starb letztendlich an den Folgen [17, 23]. Nichtsdestotrotz gibt es auch positive Entwicklungen zu verzeichnen: In Fällen von Intubationen oder Tracheotomien, bei denen das Personal ausreichende Schutzbekleidung getragen hatte, kam es nicht zu Sekundärinfektionen [7, 24, 30, 36]. Dies untermalt, was wir und andere bereits immer wieder betonen: Wenn Vorsichtsmaßnahmen korrekt befolgt werden, dann können auch bei Hochrisikoprozeduren Infektionen des medizinischen Personals effektiv vermieden werden.

Während bereits einige Publikationen zur Tracheotomie selbst existieren, gibt es bisher nur vergleichsweise wenig Information über die weiterführende stationäre Versorgung des Tracheostomas. Wir haben mithilfe unserer kleinen Studie die Gefahr der Bildung von Tröpfchen und Tröpfchenkernen bei verschiedenen Szenarien der Tracheostomapflege gemessen. Wir konnten dabei demonstrieren, dass insbesondere das Einsetzen der Trachealkanüle bei wachem Patienten eine starke Tröpfchenentwicklung im großen Radius zu beobachten war.

Zu unseren Versuchen muss kritisch bemerkt werden, dass kleinste Aerosole nicht dargestellt und gemessen wurden. Zudem ist, wie wir wissen, die Hustenneigung und -intensität stark patientenabhängig. Kernpunkt der Ergebnisse ist sicherlich, dass der Kanülenwechsel bei wachen Patienten von den ausgewählten Szenarien der am höchsten risikobehaftete ist und nur im Notfall durchgeführt werden sollte. Bei Einsetzen der Kanüle ist auch hier auf einen bereits aufgesetzten HME-Filter zu achten, um die Tröpfchenfreisetzung zu minimieren. Inhalationen sind wichtig für die Durchgängigkeit der Kanüle, sollten jedoch vor einem geplanten Wechsel so lange wie möglich ausgesetzt werden.

Ein weiterer wichtiger Punkt ist auch der Zeitverlauf der Infektiosität der SARS-CoV-2-positiven Patienten. Hierbei könnte es relevant sein, zwischen kritisch kranken, intensivpflichtigen Patienten, die z. B. für eine verbesserte Weaningsituation tracheotomiert worden sind, und SARS-CoV-2-positiven Patienten, die aus anderen medizinischen Gründen ein Tracheostoma erhalten haben oder bereits zuvor hatten, zu unterscheiden. Letztere Patienten können potenziell auch eine milde Symptomlast zeigen. Diese Unterscheidung beruht auf der Vermutung, dass sich die Zeitverläufe der Infektiosität zwischen milden und kritisch kranken SARS-CoV-2-positiven Patienten unterscheiden könnten: Bei milden Verläufen war das Virus bis zu 4 Tage (Abstrich) bzw. 8 Tage (Lungensekret) nach Symptombeginn anzüchtbar [39]. Auch das Robert Koch-Institut geht von einer durchschnittlichen Infektiosität von ca. 10 Tagen aus [21]. SARS-CoV-2-positive Patienten mit milden Verläufen blieben durchschnittlich 24,7 Tage im Krankenhaus [33]. Bei schweren bzw. kritischen Verläufen wurde der Krankenhausaufenthalt durchschnittlich mit 3–6 Wochen angegeben [44]. Ebenso verlängerte sich bei diesen Patienten die Dauer der Nachweisbarkeit der Viruslast in der PCR. Bei schweren Verläufen sind hier z. B. Virusnachweise von durchschnittlich 20 Tagen nach Symptombeginn beschrieben worden [47]. Allerdings sind diese Daten unter Vorbehalt zu betrachten, da täglich neue Erkenntnisse gewonnen werden und sich diese ersten Schlussfolgerungen später wieder ändern können.

Bezüglich der Definition der Infektiosität ist zu beachten, dass ein positiver PCR(Polymerasekettenreaktions)-Test nicht unbedingt einer Infektiosität entspricht. Eine Infektiosität liegt prinzipiell vor, wenn sich das aus Abstrichen oder Sekret gewonnene Virus in Zellkultur vermehren kann. Der PCR-Test detektiert jedoch auch die verbleibenden Reste des Virus, die sich nicht mehr vermehren können. Die Detektionsgrenze liegt laut FDA bei ca. 136 Viruskopien pro Milliliter Probe [26]. Wenn der Patient seit 48 h keine Symptome mehr aufweist und im Abstand von 24 h 2 negative PCR-Tests aufweist, gilt er als SARS-CoV-2-negativ [27]. Zum anderen gilt der Patient als SARS-CoV-2-negativ, wenn eine Serokonversion im Blut nachzuweisen ist. Erste Studien beschreiben eine Serokonversion bei milden und moderaten Fällen nach ca. 12 Tagen [39].

Die Indikation zum Kanülenwechsel muss kritisch gestellt werden. Wenn medizinisch vertretbar, sollte der Kanülenwechsel idealerweise erst nach Feststellung der SARS-CoV-2-Negativität durchgeführt werden. Sollte dies nicht möglich sein, so verweisen wir auf die oben genannten Vorschläge zum Ablauf des Kanülenwechsels sowie auf die PSA. Je nachdem, ob die Tracheotomie in einer frühen oder späten Phase der Erkrankung durchgeführt worden ist, ist eine interindividuelle Abwägung bezüglich des Zeitpunkts des Kanülenwechsels nötig. Bezüglich des geeigneten Zeitpunkts der Rückverlegung sollte zwischen einer frühen Rückverlegung zur Verminderung der Tröpfchenbildung bei der Kanülenpflege und einer späten Rückverlegung, wenn der Patient SARS-CoV-2-negativ getestet worden ist, abgewogen werden. Eigene Erfahrungen haben hier gezeigt, dass die meisten Patienten mit schweren Verläufen ein protrahiertes Weaning benötigen und bereits SARS-CoV-2-negativ getestet worden waren, als die Frage nach einer Rückverlegung des Tracheostomas gestellt wurde.

Die hier diskutierten Vorsichtsmaßnahmen zur Tracheostomapflege und ihre gefahrlose Durchführbarkeit im stationären Alltag sind leider oft noch immer von der fehlenden Verfügbarkeit der benötigten PSA abhängig. Bis dahin gilt, dass nur eine minimale Anzahl der Mitarbeiter diese Hochrisikoaufgabe übernehmen sollte und eine entsprechende Schulung des Personals vorab gewährleistet sein muss.

## Fazit

In dieser für medizinisches Personal schwierigen Zeit, welche geprägt ist durch erhöhte Belastung im Arbeitsalltag mit SARS-CoV-2-positiven Patienten und der potenziellen Gefahr der eigenen Ansteckung, sind die ersten Erfolge zum Schutz der Mitarbeiter ein ermutigendes Zeichen [12]. Diese Studie bietet sowohl Hintergrundinformation zur gegenwärtigen Situation von tracheotomierten COVID-19-Patienten als auch eine Übersicht zur Risikominimierung mit dieser Patientengruppe. Dennoch können Restrisiken auch bei Beachtung der Empfehlungen nicht ausgeschlossen werden.

## Footnotes

1 Marchioni D, Personal communication, April 2020.
